# Impaired time-distance reconfiguration patterns in Alzheimer's disease: a dynamic functional connectivity study with 809 individuals from 7 sites

**DOI:** 10.1186/s12859-022-04776-x

**Published:** 2022-07-14

**Authors:** Kai Du, Pindong Chen, Kun Zhao, Yida Qu, Xiaopeng Kang, Yong Liu, the Multi-center Alzheimer Disease Imaging Consortium, Xi Zhang, Yuying Zhou, Ying Han, Qing Wang

**Affiliations:** 1 School of Artificial Intelligence, University of Chinese Academy of Sciences, Beijing, China; 2 Brainnetome Center and National Laboratory of Pattern Recognition, Institute of Automation, Chinese Academy of Sciences, Beijing, China; 3 School of Artificial Intelligence, Beijing University of Posts and Telecommunications, Beijing, 100876 China; 4 Beijing Advanced Innovation Centre for Biomedical Engineering, School of Biological Science and Medical Engineering, Beihang University, Beijing, China

**Keywords:** Time distance nodal connectivity diversity, Dynamic functional connectivity, Network reconfiguration, Multicenter, Alzheimer's disease

## Abstract

**Background:**

The dynamic functional connectivity (dFC) has been used successfully to investigate the dysfunction of Alzheimer's disease (AD) patients. The reconfiguration intensity of nodal dFC, which means the degree of alteration between FCs at different time scales, could provide additional information for understanding the reconfiguration of brain connectivity.

**Results:**

In this paper, we introduced a feature named time distance nodal connectivity diversity (tdNCD), and then evaluated the network reconfiguration intensity in every specific brain region in AD using a large multicenter dataset (N = 809 from 7 independent sites). Our results showed that the dysfunction involved in three subnetworks in AD, including the default mode network (DMN), the subcortical network (SCN), and the cerebellum network (CBN). The nodal tdNCD inside the DMN increased in AD compared to normal controls, and the nodal dynamic FC of the SCN and the CBN decreased in AD. Additionally, the classification analysis showed that the classification performance was better when combined tdNCD and FC to classify AD from normal control (ACC = 81%, SEN = 83.4%, SPE = 80.6%, and F1-score = 79.4%) than that only using FC (ACC = 78.2%, SEN = 76.2%, SPE = 76.5%, and F1-score = 77.5%) with a leave-one-site-out cross-validation. Besides, the performance of the three classes classification was improved from 50% (only using FC) to 53.3% (combined FC and tdNCD) (macro F1-score accuracy from 46.8 to 48.9%). More importantly, the classification model showed significant clinically predictive correlations (two classes classification: R = −0.38, *P* < 0.001; three classes classification: R = −0.404, *P* < 0.001). More importantly, several commonly used machine learning models confirmed that the tdNCD would provide additional information for classifying AD from normal controls.

**Conclusions:**

The present study demonstrated dynamic reconfiguration of nodal FC abnormities in AD. The tdNCD highlights the potential for further understanding core mechanisms of brain dysfunction in AD. Evaluating the tdNCD FC provides a promising way to understand AD processes better and investigate novel diagnostic brain imaging biomarkers for AD.

## Background

Alzheimer's Disease (AD) is one of the most common neurodegenerative diseases with impaired multi-functions, including cognition decline, impaired memory, behavioral disorders, and emotional changes [1, 2]. As a complex adaptive system, the AD brain shows dysfunctions when it integrates diverse cognitive processes into a coherent whole as a function of changing across various time scales [3–6]. Furthermore, considering the brain dynamics, these abnormalities of functional integration in AD are associated with the aberrant transitions between different active states [7]. Hence, understanding the AD-associated impaired brain alteration pattern in different time scales would be very important for understanding the mechanism of cognitive declines in AD [8].

Functional connectivity (FC) is defined as the temporal correlation of neuronal activity in anatomically isolated regions of the brain, which can be used to explain the synchronizations between spatially remote areas of the brain [9, 10]. FC represents the correlation between time series obtained in many ways, such as functional magnetic resonance imaging (fMRI). The brain dynamically reconfigures its functional organization to support the common cognitive ability of the brain, of which FC is one of the most common measures of the brain's active organization[11, 12]. The concept of static FC assumes the spatial and temporal stationarity of functional synchronization when a subject is lying in the scanner for around 6–10 min [13, 14]. However, this assumption ignored the fast conversion between different mental states [15], which had different effects on FC [16]. It is well accepted that the brain dynamically reconfigures to support the information transformation in the daily cognitive task. Previous studies also demonstrated that the ability impairment is associated with the brain dynamic reconfigures, in other words, the transition between different dynamic FC (dFC) states rather than the statical model [17, 18]. Thus, the dFC allowed us to investigate the brain as a dynamic functional network to capture the different brain states and/or the associated impaired activities [19–21]. Quantitatively depicting the abnormal dynamic functional reconfigures and how they lead to dementia or specific cognitive impairment is essential for better understanding the abnormalities of dFC in AD [22]. One way is to investigate the brain network dynamics by calculating independent components of dFC and defining a series of brain states. And the transitions between different brain states were used to assess whether or not brain activity is impaired in certain diseases [23–29].

Although dynamic FC has been proposed and widely used to analyze the reconfiguration pattern of the dynamic functional network in AD [17, 18, 30], the strength of the dynamic network configuration of rs-fMRI has not been well investigated yet. Thus, quantitatively depicting the abnormal dynamic functional reconfigures and how they lead to dementia or specific cognitive impairment is essential for better understanding the FC’s abnormality in AD. Hence, the main aim of the present study is to investigate whether there are abnormal nodal FC reconfigurations in some regions at some specific frequencies and the clinical relevance of that abnormality in AD.

**Fig. 1:**
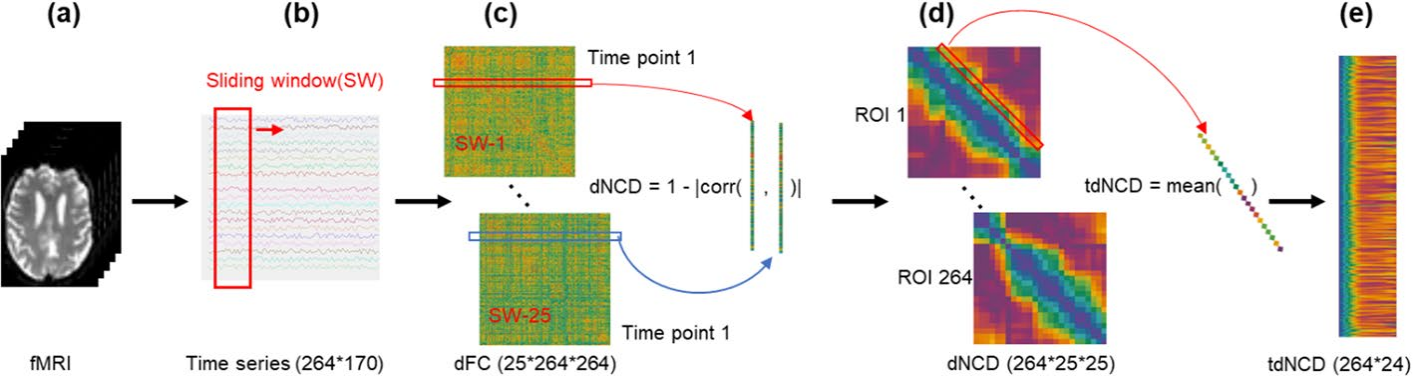
Schematic pipeline for computing tdNCD for each subject. **a** The fMRI images( Each fMRI had 170 time points). **b** The mean time series (264 × 170) which was calculated based on the Power’s atlas. The sliding window technique was performed to calculate dFC. **c** The dFC matrix (25 × 264 × 264). **d** The dNCD was obtained from the dFC according to the formula (2). **e** The tdNCD was calculated from the mean of dNCD at each time distant according to the formula (3)

**Fig. 2:**
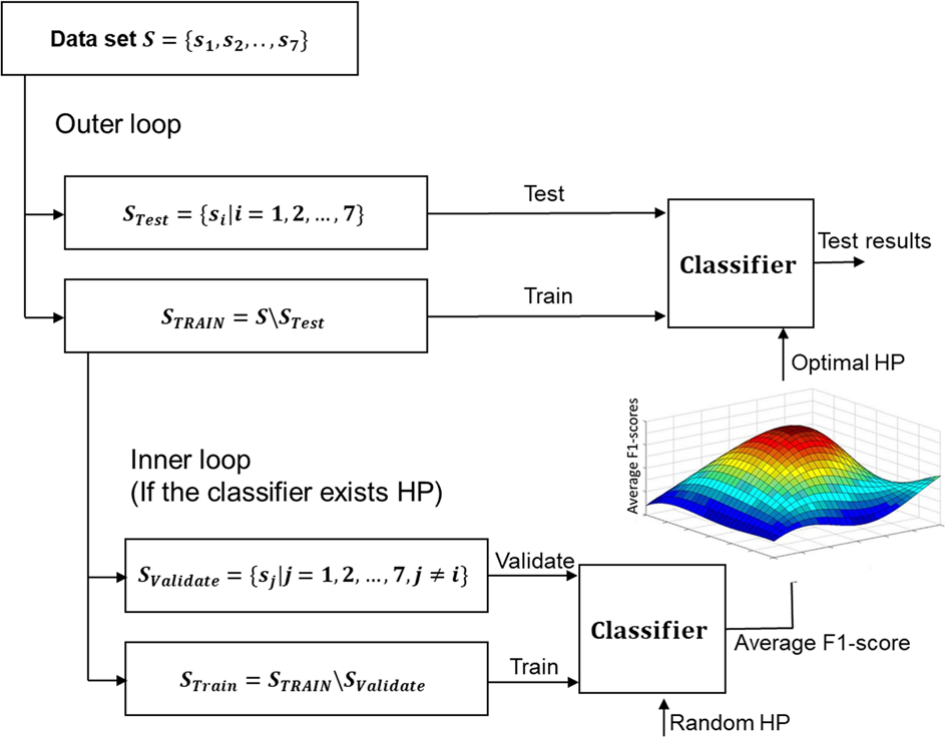
The strategy of the train and test framework for these classifiers. One dataset was chosen as the testing set in the outer loop, and the other six were used to optimize the hyperparameters and train the models. If the classifier needs to select the hyperparameter (HP), we took two steps to train the model to determine the optimal HPs. Specifically, there are two HPs (iteration times of input data and learning rate of the Adam optimizer) for FCnet. Meanwhile, we used the leave-one-site-out strategy to validate the robustness of the models

In this study, we introduced the time distance nodal connectivity diversity (tdNCD) to quantify the strength of nodal FC reconfiguration at different time scales and compared the changes tdNCD in AD for each site [12]. Then, meta-analyses were performed to combine data from the individual scanners and test for differences in tdNCD between AD patients and the normal controls (NCs) (Fig. 1). Additionally, we used four layers fully connected neural network (FC-net) to investigate the classification performance of the tdNCD (Fig. 2). Our results indicate that AD patients are characterized by aberrant tdNCD in several core networks, such as the default mode network. This provides a new perspective to understand the neuropathological mechanism underlying AD and may be a useful imaging biomarker for identifying AD.

## Results

### Demographic and neuropsychological characteristics

There were no significant differences in the sex of the NC, MCI, and AD subjects totally (*P* = 0.658, Chi-squared test). But the age of the MCI and AD subjects exhibited significantly higher than those of the NC subjects (*P* = 0.011, one-way ANOVA). Moreover, the subjects of the MCI and AD groups showed significantly lower scores on the MMSE than those of the NC group (*P* < 0.001, one-way ANOVA) (Table 1). The fMRI scanner and image-acquisition protocol information in Table 2.

**Table 1:** Demographic and neuropsychological data of participants. Chi-squared tests were used for gender comparisons; one-way ANOVA was performed for age and MMSE comparisons

	NC	MCI	AD	*p*
N (809)	257	257	295	–
Sex(M/F)	153/104	143/114	172/123	0.658
Age	66.93 $\pm$ 6.83	68.56 $\pm$ 8.91	68.89 $\pm 8.27$	0.011
MMSE	28.52 $\pm$ 1.64	25.14 $\pm$ 3.39	16.56 $\pm$ 6.02	< 0.001

**Table 2:** The fMRI scanner and image-acquisition protocol information of the in-house dataset

	Site 1	Site 2	Site 3	Site 4	Site 5	Site 6	Site 7
Field of strength (T)	3.0	3.0	3.0	3.0	3.0	3.0	3.0
Brand	Siemens Skyra	GE Signa HDx	Siemens Trio Tim	Siemens Verio	Siemens Trio Tim	Siemens Trio Tim	Siemens Skyra
Number of head coil channels	20	8	20	8	12	8	20
Protocol name	EPI	EPI	EPI	EPI	EPI	EPI	EPI
Repetition time (s)	2	2	2	2	2	2	2
Echo time (ms)	30	30	25	30	40	30	30
Flip angle	90	90	90	90	90	90	90
Field of view	220 × 220	220 × 220	240 × 240	220 × 220	256 × 256	220 × 220	220 × 220
Matrix	64 × 64	64 × 64	64 × 64	64 × 64	64 × 64	64 × 64	64 × 64
Slice number /thickness (gap)	36 / 3 (0.9)	30 / 3 (1)	30 / 3 (1)	36 / 3 (0.99)	28 / 4 (1)	32 / 3 (0.99)	36 / 3 (0.9)
Scan duration (s)	480	400	360	360	478	360	480

### Group difference between NC and AD

**Fig. 3:**
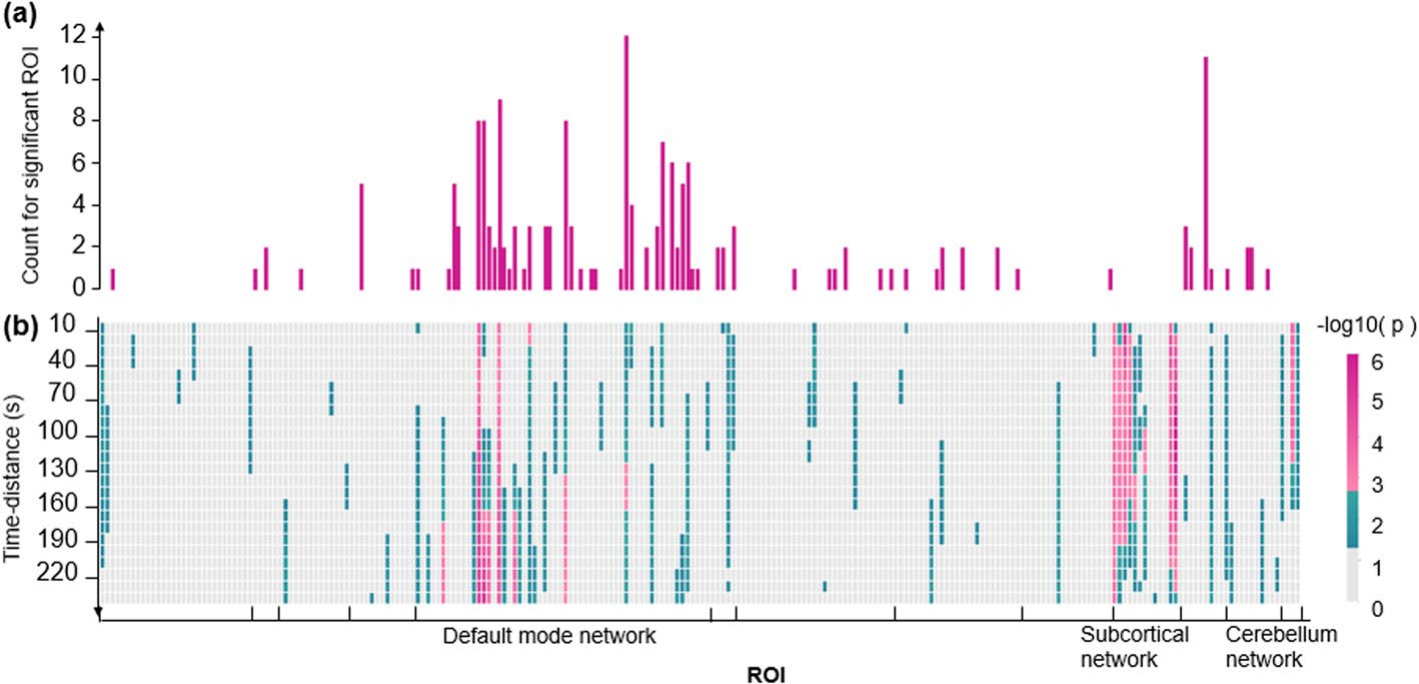
Results of differences FC or tdNCD between AD and NC with meta-analysis. **a** The number of the abnormal FCs (Bonferroni correction, N = 264). The bar length means the number of significantly different FCs between one ROI and the other ROIs. **b** The distribution of the altered tdNCD in AD. The *p*-value was obtained by meta-analysis in 7 sites. The significant threshold of the *p*-value is 0.0021 (= 0.05/24, Bonferroni correction)

The tdNCD significantly changes in the default mode network (DMN), the SCN, and the cerebellum network (CBN) at different time distances in AD groups compared with NC (*P* < 0.05, Bonferroni correction with N = 24) (Fig. 3). Specifically, tdNCD was higher in the DMN (the inferior parietal lobule, precuneus, parieto-occipital sulcus, superior frontal gyrus, middle temporal gyrus, etc.) in AD than NC. Meanwhile, the tdNCD was lower in the SCN (the thalamus, basal ganglia, inferior temporal gyrus, orbital gyrus, inferior occipital gyrus) and the CBN (area of the cerebellum) in AD than in the NC (Fig. 3a). Group differences of FC between NC and AD were analyzed using the meta-analysis as in our previous study [31]. After Bonferroni correction with N = 264, the number of significantly different FC between one ROI and the other ROIs is shown in Fig. 3a. And if the number of significant FC of one ROI was more than zero, the ROI was considered to be significantly different. In contrast to the FC, the tdNCD provides more information about the impaired functional connectivity in AD. For example, one region in the dorsal parieto-occipital sulcus mainly showed the changes in AD at the long-time distances (170 s-240 s). In contrast, another area at the lateral superior frontal gyrus showed significant differences in AD at the short time distance (0–20 s) (Fig. 3).

**Fig. 4:**
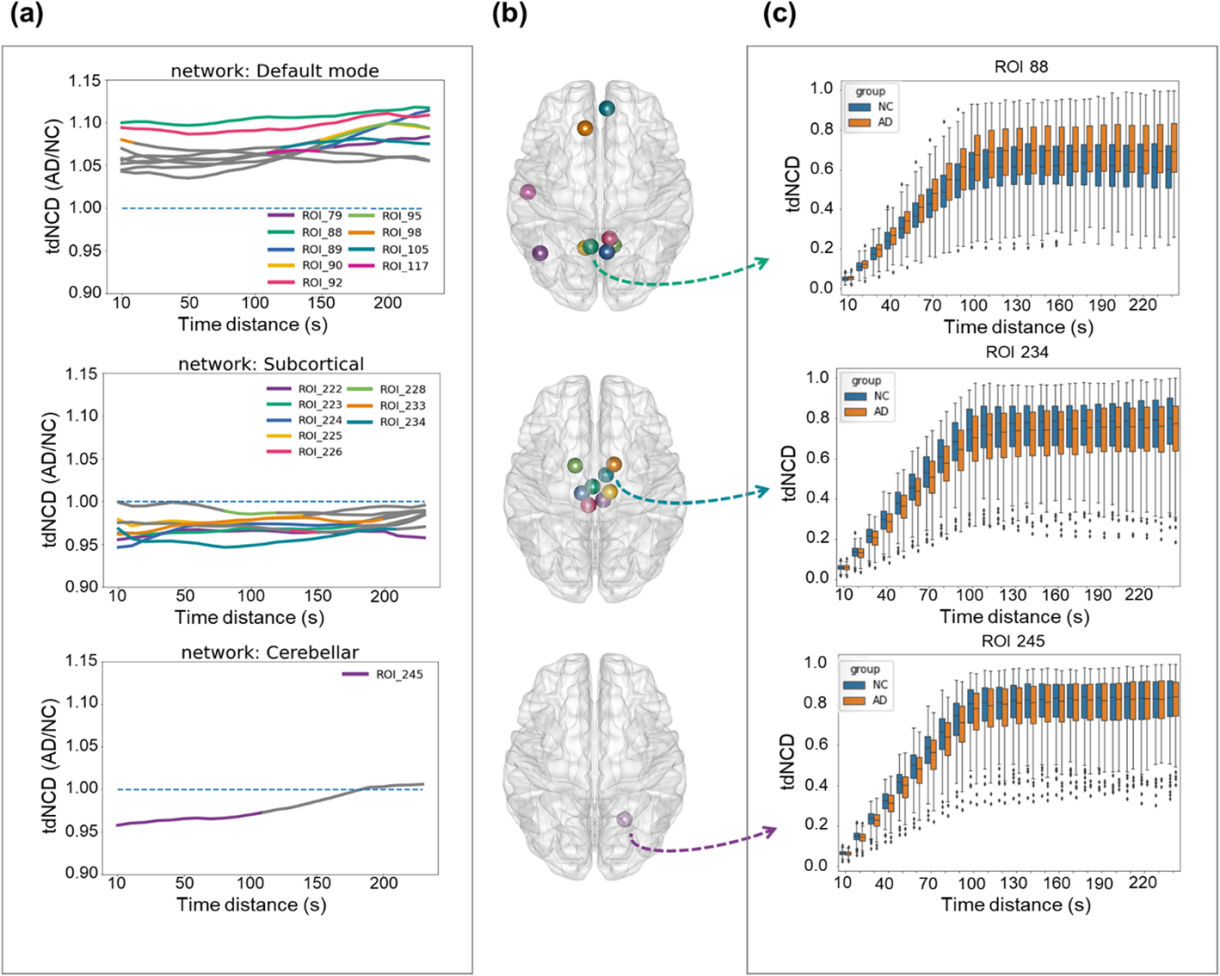
Results of differences analysis based on the tdNCD. **a** The time distance associated altered patterns of the tdNCD (AD/NC) in three representative networks. The gray line represents that the tdNCD (AD/NC) is not significant at the specific time distance of the related ROI. **b** Scatter plots of the ROIs. **c** Boxplots of the time distance associated patterns of the tdNCD in three represent ROIs in the AD and NC groups. The error bar represents the standard deviation

Additionally, the value of tdNCD increased with the short time distance (time distance < 100 s) and remained relatively stable at the long-time distance (time distance > 100 s) (Fig. 4). The tdNCD with a short time distance represented network configuration flexibility, and the tdNCD with a long-time distance meant the tendency to lose network stability. Although tdNCD with long time distance was calculated by fewer data, the uniform difference obtained by many subjects after meta-analysis can reflect the network reconfiguration.

### Classification based on FC and tdNCD

**Fig. 5:**
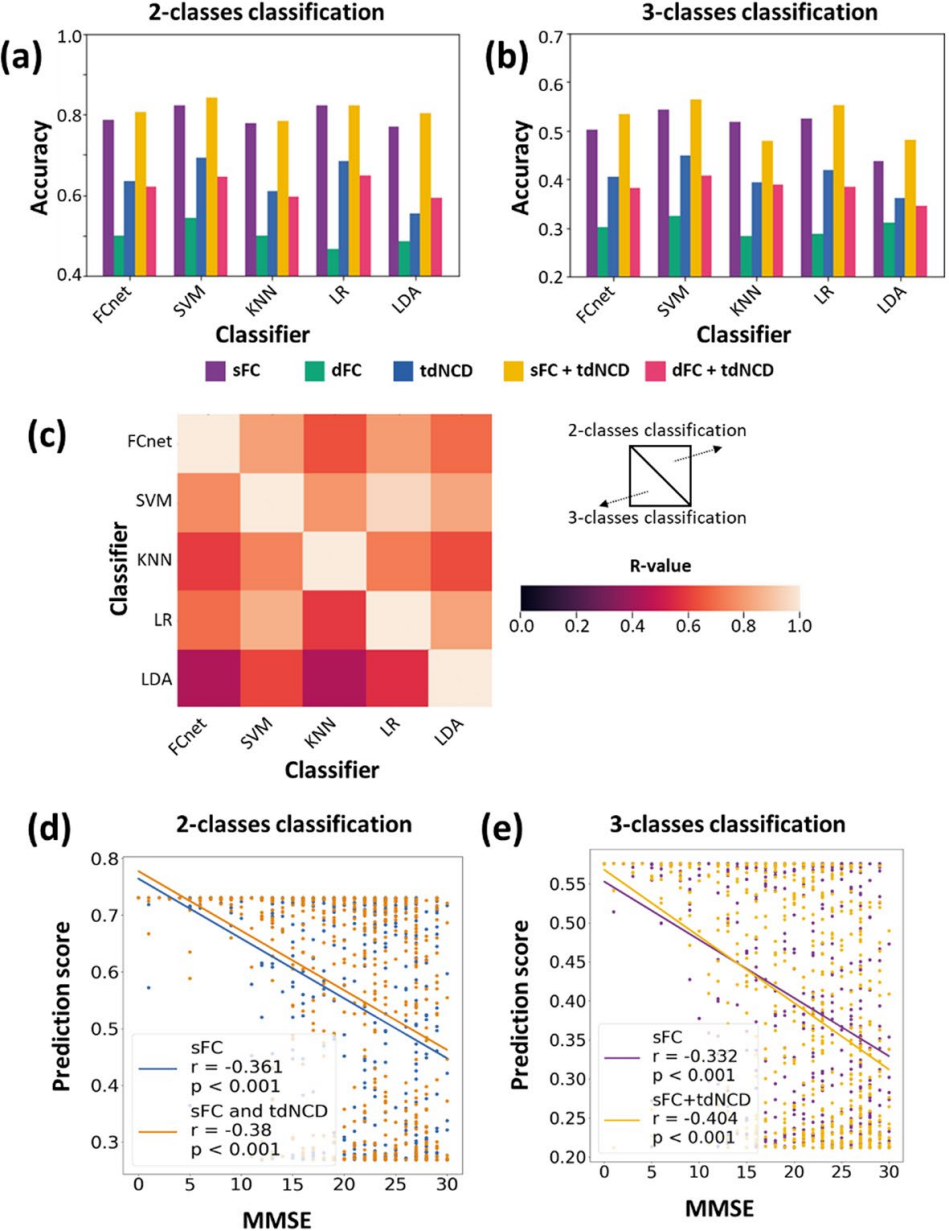
Results of classification analysis by using five classifiers with different input features. **a** The accuracy of these two-class classification models. **b** The accuracy of these three-class classification models. **c** The R-values of correlation between the decision scores associated with the AD group from these classification models. The R-values located in the top right were from the two-class classification models. The R-values on the left bottom were from the three-class classification models. **d** The correlation between subjects’ MMSE and decision scores from a two-class classifier. **e** The correlation between subjects’ MMSE and decision scores from a three-class classifier

The classification results showed that the tdNCD has a contribution to improving the effect of AD identification from NC (Accuracy: from 78.2% to 81%, *P* = 0.028; Sensitivity: from 76.2 to 83.4%, *P* = 0.043; Specificity: from 80.6 to 76.5%, P = 0.198; F1-score: from 77.5% to 79.4%, *P* = 0.086) (Table 3). Our results showed significant negative correlations between the individual decision scores and cognitive ability (measured by MMSE) in the AD and MCI subjects (R = −0.38, *P* < 0.001), which was improved than that only using FC (R = −0.361, *P* < 0.001) (Fig. 5d).

**Table 3:** The results of two classes classification

Classifier	Features	ACC (%)	SEN (%)	SPE (%)	F1-score (%)
FCnet	sFC	78.2	76.2	80.6	77.5
	sFC + tdNCD	81*	83.4	76.5*	79.4*
SVM	sFC	82.3	85.6	76.6	83.2
	sFC + tdNCD	84.2	86.2	81.5*	85.2*
KNN	sFC	77.9	69.6	85.8	76.2
	sFC + tdNCD	78.4	73.5*	83.9	78.4
LR	sFC	82.3	82.5	81.8	82.9
	sFC + tdNCD	82.1	81.1	81.5	82.1
LDA	sFC	77.0	79.6	72.7	78.6
	sFC + tdNCD	80.2*	84.4*	73.8	81.9*

*Means prediction results have significant improvement when tdNCD was added (*p* < 0.05, paired-sample t-test)

Besides, we conducted a similar pipeline to analyze the classification performance for NC, MCI, and AD individuals. The results showed that there was a significant improvement in accuracy (from 50% to 53.3, *P* = 0.01) and macro F1-score (from 47.2 to 49.1%, *P* = 0.034) when tdNCD was added to the classifier (Table 4). The output of the three-class classification model was a 1 × 3 vector. The three numbers represented the probability of belonging to the NC, MCI, or AD group, respectively. A significant correlation between prediction score and MMSE score was also obtained in the 3-class classification model (R = −0.404, *P* < 0.001), which was improved than that only using FC (R = −0.332, *P* < 0.001) (Fig. 5e).

**Table 4:** The results of three classes classification

Classifier	features	ACC (%)	SEN (%)	SPE (%)	F1-score (%)
FCnet	sFC	50	50.6	75.3	46.8
	sFC + tdNCD	53.3*	52.0	76.0	48.9*
SVM	sFC	54.2	54.3	77.1	50.7
	sFC + tdNCD	56.3*	56.1	78.1	53.4*
KNN	sFC	51.8	51.8	75.9	48.8
	sFC + tdNCD	47.9	47.9	73.9	45.7
LR	sFC	52.4	52.4	76.2	50.3
	sFC + tdNCD	55.1*	55.1*	77.5*	52.5
LDA	sFC	43.8	43.8	71.9	42.7
	sFC + tdNCD	48.1*	48.1*	74.0*	45.8*

*Means prediction results have significant improvement when tdNCD was added (*p* < 0.05, paired-sample t-test)

The classification results are robust for different classification models, whether in the two-class task (Table 3) or the three-class tasks (Table 4). Generally, the combination of tdNCD and sFC/dFC could improve the classification performance rather than only based on sFC/dFC. Furthermore, the decision value of the testing datasets showed high consistency among different pairs of classifiers (Fig. 5c).

**Fig. 6:**
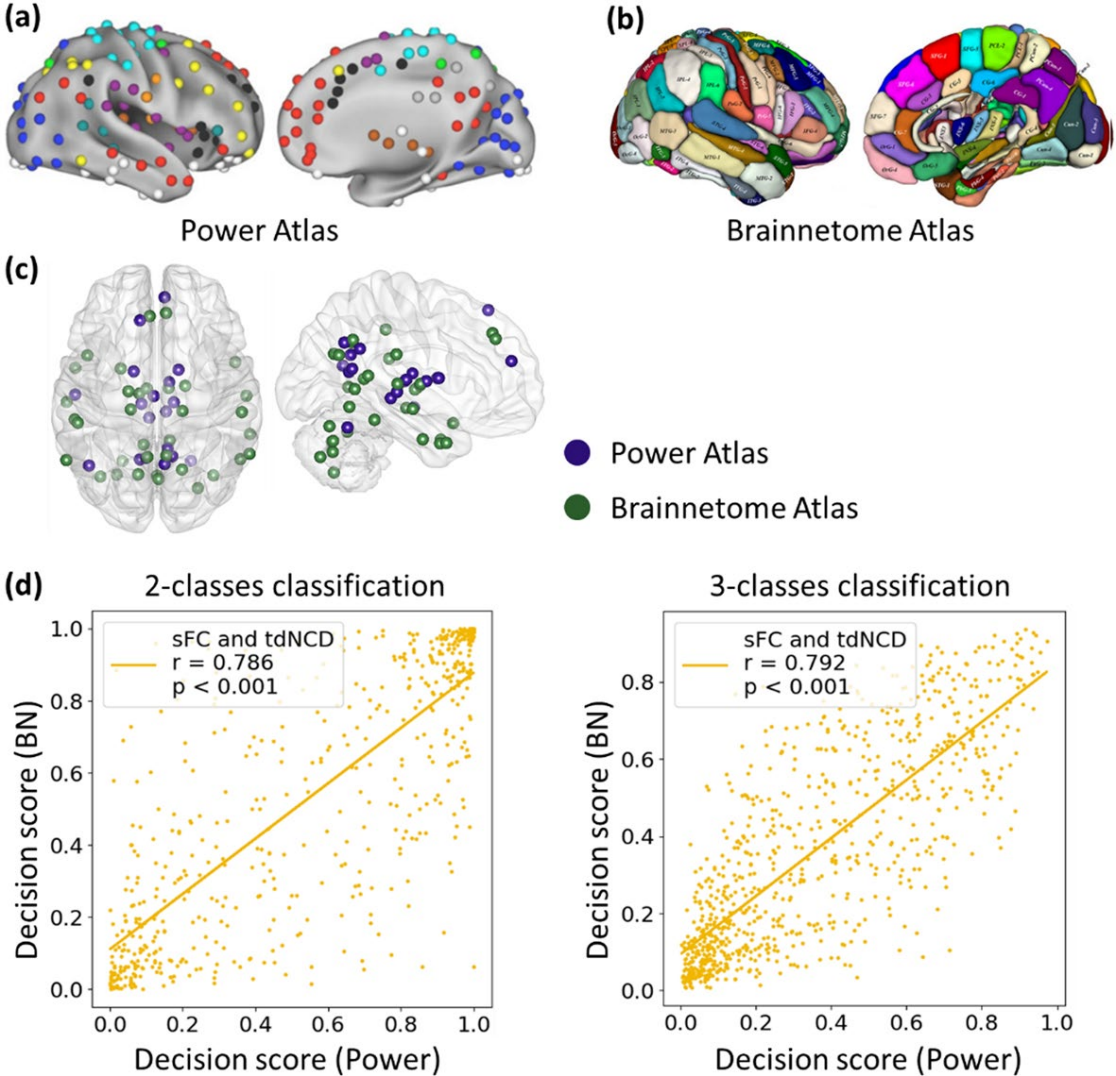
The results of the replicability experiment by using the BN Atlas. **a** The Power atlas [51]. **b** The Brainnetome (BN) Atlas [63]. **c** The results of significantly different ROIs mapping to the whole brain by using the same process introduced in this paper. **d** The correlation between the decision scores associated with the AD group from the two-class classification model using the Power Atlas and the two-class classification model using the BN atlas. **e** The correlation between the decision scores associated with the AD group from the three-class classification model using the Power Atlas and the three-class classification model using the BN atlas

Figure 6 shows the tdNCD of nodes that showed a consistent significant difference between AD and NC based on two atlas, there was located in the superior frontal gyrus, the temporal gyrus, the parietooccipital sulcus, the precuneus, the thalamus, the cerebellum, the basal ganglia based on Power Atlas, and in the superior frontal gyrus, the temporal gyrus, the inferior parietal lobule, the precuneus, the thalamus, the cerebellum, the parahippocampal gyrus, the posterior cingulate gyrus based on Brainnerome Atlas. Besides, the decision value of the testing data based on Power Atlas was significantly correlated with that based on Brainnetome Atlas, whether in the two-class task (R = 0.786, *P* < 0.001) or in the three-class task (R = 0.792, *P* < 0.001). Therefore, the result can be repeated under the different brain atlases or parcellation schemes.

## Discussion

The present study provided a novel method to represent the change of nodal dFC at different time scales in AD, suggesting that the connectivity and the dynamics of activity altered in several brain networks in AD. Furthermore, the tdNCD provides benefits for classifying AD from normal controls. Moreover, the individuals’ decision scores of the classification significantly correlated with cognitive impairment in the patients' groups which means the individual variation in the severity of these abnormalities predicts the severity of cognitive impairment. These findings emphasize the importance of nodal FC reconfiguration and the potential of tdNCD to provide a robust imaging signature of AD.

The sFC is an essential biomarker for studying the mechanism of various neurodegenerative diseases [6]. And there have been numerous research efforts using FC to explain the impairments of the brain in AD. Specifically, convergent evidence suggested that the AD patients showed a pattern of widespread dysconnectivity in the brain [32–34]. Our previous study has found that brain activity was reduced in the DMN and increased in the SCN in AD patients [31, 35]. And the strength of brain activity in these networks was significantly associated with the impairments decline [31]. The DMN will be activated when we pay no attention to the external environment [36]. Previous studies have demonstrated that the aberrant FCs in AD patients were related to the pathophysiology of AD. For example, the abnormal FC in the DMN was linked with the deposition of the amyloid-beta (Aβ), and is further associated with spatial and autobiographical memory[36].

Recently, several studies have shown that the states of dFC were highly alterable, and the AD patients tended to stay in low inter-network interactions [6, 17, 18]. The present study found that the dFC increased in the DMN and reduced in the SCN in AD patients. This is supported by the evidence that AD patients are associated with disrupted functional connectivity in the DMN. The tdNCD represented the reconfiguration intensity of the dFC network. The increase of tdNCD in the DMN exhibited disrupted DMN integrity [37], which induced the impaired ability to integrate and share information in AD [38]. On the other hand, a decrease of the tdNCD in the SCN and CBN indicated enhanced inner stability in these networks [37, 39]. These changes involved some hub regions, which played an essential role in distinct interconnecting areas and integrating brain function [40–42]. And the impairments may lead to the abnormal increase of tdNCD in the DMN. On the other hand, the accumulation of Aβ in the SCN and CBN [43, 44] may be related to the decrease of FCs in the different regions, the compensatory decrease of inner FCs, and the tdNCD decrease in these regions.

As discussed in several previous studies [31, 45–49], cross-site validation is crucial for optimizing valid biomarkers and is particularly important for translational medicine. Hence, the classification analysis with independent site cross-validations to predict diagnostic status provides shreds of evidence that tdNCD can provide additional information as imaging biomarkers for AD. However, tdNCD itself does not have a good performance for classification. The remarkably reduced classification performance in the MCI group might be due to the data heterogeneity between different sites. These heterogeneities may come from the various scanning devices or the diagnostic criteria for early MCI. Furthermore, the increase in the correlations between the decision scores and clinical scores indicated the ability to use dynamic brain activity to track disease progression [31].

There were some limitations to this study. Firstly, the window size from the sliding window technique was used to calculate dFC, hampering our efforts to analyze the nodal dFC at a short time distance. Secondly, the analysis of nodal dFC was just at the network level, and it was not precise enough. Thirdly, variations in the acquisition methods and cross-sectional examination of neuropsychological performance precludes us from making firm conclusions about group differences. Fourthly, the MCADI dataset does not have pathological measures, like Abeta and Tau; it might result in a few misdiagnose in MCI.

## Conclusion

The present study provides a simple way to explore the stable abnormality of dFC in AD. The results showed that the tdNCD can be used to analyze the stability of dFC in specific brain regions and contains additional information for understanding the alteration of brain activity in AD. Furthermore, investigating the stability of the dynamic connectivity at different time scales can provide comprehensive insights into the functional reconfiguration, advancing our knowledge of cognitive decline in AD.

## Material and method

### Datasets

The present study is an extended investigation of our previous results, and all analyses were conducted in an in-house Multi-Center Alzheimer's Disease (MCAD) dataset. This dataset includes 809 individuals (AD = 295, mild cognitive impairment (MCI) = 257, NC = 257) with resting-state fMRI were acquired from 7 different MRI scanners. In addition, we provided demographic and neuropsychological information in Table 1, and the data can be found elsewhere in our previous study [31]. Finally, we used the Mini-Mental State Exam (MMSE) score to measure subjects’ cognitive capacity.

### Image preprocessing and dynamic functional connectivity matrix calculation

All fMRI images were obtained from 3.0 T MR scanners. The shortest scan duration was 360 s with repetition time (TR) = 2 s. Thus, we used the first 360 s of all fMRIs in this paper. The first 10 images were discarded to allow for magnetization equilibrium, we got the fMRIs with 170 time points. As described in our previous study [31], the resting-state fMRI (rs-fMRI) scans were preprocessed using the Brainnetome Toolkit (http://brant.brainnetome.org) [50] with the following steps (1) slice timing correction; (2) realignment to the first volume; (3) spatial normalization to Montreal Neurological Institute (MNI) space at 2 mm × 2 mm × 2 mm; (4) regression of nuisance signals, including linear trends, six motion parameters, and their first-order differences, and signals representing white matter and cerebrospinal fluid; (5) temporal bandpass filtering (0.01–0.08 Hz) to reduce high-frequency noise. Detailed descriptions can be found elsewhere in our previous study [31]. After these preprocessing steps, one 264(ROIs) × 170(time points, TR = 2 s) matrix was obtained for each subject. Then, dFC matrices were calculated based on 264 predefined regions of interest (ROIs) across the entire brain [51] as follows:

1 $$\begin{array}{*{20}c} {dFC_{i}^{m,n} = corr\left( {TS_{i,i + W}^{m} ,TS_{i,i + W}^{n} } \right)} \\ \end{array}$$

where *W* represents the window size (= 100 s), $TS_{i,i + W}^{m}$ represents the time series between timepoint *i* and timepoint $i + W$ in region $m$, $corr\left( {TS_{i,i + W}^{m} ,TS_{i,i + W}^{n} } \right)$ represents the Pearson’s correlation coefficient between $TS_{i,i + W}^{m}$ and $TS_{i,i + W}^{n}$, $dFC_{i}^{m,n}$ represents the dFC between region *m* and region *n* at timepoint *i*.

### Nodal connectivity diversity of dynamic functional connectivity

The NCD was usually used in mathematics and signal processing and has been used to describe the distance between connection profiles of a node in two mental states (e.g., resting or performing tasks [28]). In that study [12], the NCD is defined as:

2 $$\begin{array}{*{20}c} {NCD_{i,j}^{n} = 1 - \left| {corr\left( {FC_{i}^{n} ,FC_{j}^{n} } \right)} \right|} \\ \end{array}$$

where $FC_{i}^{n}$ represents an *n*-th row of FC matrix in mental state *i*, $corr\left( {FC_{i}^{n} ,FC_{j}^{n} } \right)$ represents the Pearson's correlation coefficient between $FC_{i}^{n}$ and $FC_{j}^{n}$.

We proposed a dynamic NCD (dNCD) index to represent the change of the dFC from one to another timepoint. The dNCD is calculated as:

3 $$\begin{array}{*{20}c} {dNCD_{i,j}^{n} = 1 - \left| {corr\left( {dFC_{i}^{n} ,dFC_{j}^{n} } \right)} \right|} \\ \end{array}$$

where $dFC_{i}^{n}$ represents an $n$-th row of FC matrix in timepoint *i*, $corr\left( {dFC_{i}^{n} ,dFC_{j}^{n} } \right)$ represents the Pearson's correlation coefficient between $dFC_{i}^{n}$ and $dFC_{j}^{n}$. As we know that the brain network dynamic alteration is relatively stable in a temporally coordinated manner. With this, the time distance NCD (tdNCD) is defined as:

4 $$\begin{array}{*{20}c} {tdNCD_{d}^{n} = \frac{{\mathop \sum \nolimits_{i}^{T - d} dNCD_{i,i + d}^{n} }}{T - d}} \\ \end{array}$$

where $dNCD_{i,i + d}^{n}$ represents n-th ROI of dNCD between timepoint *i* and timepoint $i + d$, *T* represents total time-distance.

The pipeline of calculating tdNCD is shown in Fig. 5. Specifically, each ROI had 170 time points. We calculated the mean values in each ROI defined by Power’s atlas from these fMRIs, and obtained the time series (264(number of ROIs) × 170(Time points)) after preprocessing. The sliding window technique (window size: 100 s, window step: 10 s) was used to calculate the dFC [52–54] from these time series. Next, we calculated the dNCD according to formula (2) from dFC and obtained the tdNCD according to formula (3). For example, in one subject's tdNCD matrix, point (x, t) represents the mean of dNCDs that have t time distance in ROI x (Fig. 5).

### Group-level statistical analysis for tdNCD

We used a two-sample two-sided t-test to verify the inter-group differences of tdNCD between NC and AD at each site. After that, a meta-analysis was used to reduce the impact of site differences. As suggested by the previous studies [31, 55], the Liptak-Stouffer z-score was used to combine p-values obtained by the two-sample two-sided t-test from the different sites, which has optimal power for combining probabilities in meta-analyses [55–57]. Specifically,, the $p_{i}$-values for each dataset were transformed into $z_{i}$-scores using the inverse standard normal distribution, that is:

5 $$\begin{array}{*{20}c} {z_{i} = \varphi^{ - 1} \left( {1 - \frac{{p_{i} }}{2}} \right)} \\ \end{array}$$

where $\varphi$ is the standard normal cumulative distribution function. Then the combined z-score was then computed using the Liptak-Stouffer formula:

6 $$\begin{array}{*{20}c} {z = \frac{{\mathop \sum \nolimits_{i}^{k} w_{i} z_{i} }}{{\sqrt {\mathop \sum \nolimits_{i}^{k} w_{i}^{2} } }}} \\ \end{array}$$

where $w_{i}$ is the square root of the sample size of dataset $i$, and $k$ is the number of datasets (here, $k$ = 7).

Under the null hypothesis, the z-scores follow the standard normal distribution. Therefore, by converting the z-scores to p-values with

7 $$\begin{array}{*{20}c} {p = \varphi \left( z \right)} \\ \end{array}$$

Specifically, we repeated 24 times at one ROI to analyze whether this ROI was significant. The Bonferroni correction was used to correct multiple comparisons across the 24-time distances (Bonferroni correction, N = 24). We also perform the same calculation for FC to compare the difference between the statistical analysis results about static FC (sFC) and tdNCD (Bonferroni correction, N = 264).

### Classification based on FC and tdNCD

In previous studies, the neural network has been successfully used to analyze the features obtained from fMRI [58–60], because of its higher efficiency for high dimensional complex features [61, 62]. And, the primary aim of this study is to explore whether the performance could be improved by combining the tdNCD and sFC than that only based on sFC. Therefore, we chose a simple 4-layers fully connected neural network (FCnet) as the classifier to analyze the classification performance of tdNCD. A two-sided two-sample t-test was used to test the significant difference between the performance of the classification model based on the combination of sFC with tdNCD and only based on sFC, as well as based on the dFC + tdNCD and only based on dFC. Additionally, to test whether the results are robust for the different classification models, we also performed the classification analysis with the other four traditional classifiers (SVM, K-nearest neighbor (KNN), Logistic Regression (LR), and Linear Discriminant Analysis (LDA)). Additionally, considering the number of these features is much larger than the number of subjects, a two-sample t-test was used to select the potentially efficient features in the training dataset. The appropriate threshold was chosen to keep the number of selected features and the number of subjects in the same order of magnitude (threshold *P* = 0.001 (sFC), threshold *P* = 0.00001 (dFC), threshold *P* = 0.05 (tdNCD)).

The classification performance was evaluated using accuracy (ACC), sensitivity (SEN), specificity (SPE), and F1-score. If the classifier needs to select the hyperparameter (HP), we took two steps to train the model to determine the optimal HPs. Specifically, there are two HPs (iteration times of input data and learning rate of the Adam optimizer) for FCnet. Meanwhile, we used the leave-one-site-out strategy to validate the robustness of the models [31, 45, 48] (Fig. 6). One dataset was chosen as the testing set, and the other six were used to optimize the hyperparameters and train the model in the outer loop. Five datasets were chosen from the six datasets as the training set in the inner loop, and the other served as validation data to find the optimal HPs. The performance of the classification model was determined in the testing dataset (Fig. 6).

We used the above strategy to test the performance of tdNCD in both of the two classes classification (NC and AD) and the three classes classification (NC, MCI, and AD). To best the generalizability of these analyzes, we have performed the whole analysis based on the BN Atlas [63].

## Data Availability

Brant is freely available as open-source software at www.brant.brainnetome.org. Data about the results of this study may be obtained through the corresponding author upon reasonable request. The datasets are available at https://github.com/YongLiuLab/MCADI/tree/master/KaiDu_tdNCD.

## Abbreviations

ACC: Accuracy
AD: Alzheimer’s Disease
CBN: Cerebellum network
dFC: Dynamic functional connectivity
sFC: Static functional connectivity
dNCD: Dynamic nodal connectivity diversity
DMN: Default mode network
FC: Functional connectivity;
fMRI: Functional magnetic resonance imaging
MCI: Mild cognitive impairment
NC: Normal control
NCD: Nodal connectivity diversity
rs-fMRI: Resting-state functional magnetic resonance imaging
SCN: Subcortical network
SEN: Sensitivity
SPE: Specificity
tdNCD: Time distance nodal connectivity diversity
